# Human newborn bacille Calmette–Guérin vaccination and risk of tuberculosis disease: a case-control study

**DOI:** 10.1186/s12916-016-0617-3

**Published:** 2016-05-16

**Authors:** Helen A. Fletcher, Ali Filali-Mouhim, Elisa Nemes, Anthony Hawkridge, Alana Keyser, Samuel Njikan, Mark Hatherill, Thomas J. Scriba, Brian Abel, Benjamin M. Kagina, Ashley Veldsman, Nancy Marín Agudelo, Gilla Kaplan, Gregory D. Hussey, Rafick-Pierre Sekaly, Willem A. Hanekom, the BCG study team

**Affiliations:** London School of Hygiene & Tropical Medicine, London, UK; Department of Pathology, Case Western Reserve University, Cleveland, OH USA; South African Tuberculosis Vaccine Initiative (SATVI), Institute of Infectious Disease and Molecular Medicine, Division of Immunology, Department of Pathology, University of Cape Town, Cape Town, South Africa; Grupo de Inmunología Celular e Inmunogenética, Sede de Investigación Universitaria, Universidad de Antioquia, Medellín, Colombia; Public Health Research Institute, Rutgers Biomedical and Health Sciences, Newark, NJ USA

**Keywords:** Tuberculosis, Vaccine, Correlates of risk, Systems biology

## Abstract

**Background:**

An incomplete understanding of the immunological mechanisms underlying protection against tuberculosis (TB) hampers the development of new vaccines against TB. We aimed to define host correlates of prospective risk of TB disease following bacille Calmette–Guérin (BCG) vaccination.

**Methods:**

In this study, 5,726 infants vaccinated with BCG at birth were enrolled. Host responses in blood collected at 10 weeks of age were compared between infants who developed pulmonary TB disease during 2 years of follow-up (cases) and those who remained healthy (controls).

**Results:**

Comprehensive gene expression and cellular and soluble marker analysis failed to identify a correlate of risk. We showed that distinct host responses after BCG vaccination may be the reason: two major clusters of gene expression, with different myeloid and lymphoid activation and inflammatory patterns, were evident when all infants were examined together. Cases from each cluster demonstrated distinct patterns of gene expression, which were confirmed by cellular assays.

**Conclusions:**

Distinct patterns of host responses to *Mycobacterium bovis* BCG suggest that novel TB vaccines may also elicit distinct patterns of host responses. This diversity should be considered in future TB vaccine development.

**Electronic supplementary material:**

The online version of this article (doi:10.1186/s12916-016-0617-3) contains supplementary material, which is available to authorized users.

## Background

Newborn vaccination with bacille Calmette–Guérin (BCG) protects infants and young children against disseminated forms of tuberculosis (TB), and to some extent, against pulmonary TB [1]. As BCG’s protective efficacy in other age groups is variable and mostly poor [1], new vaccines against TB are needed. An incomplete understanding of immunological mechanisms underlying protection against TB hampers vaccine discovery and development. Our aim was to define host correlates of prospective risk of TB disease following BCG vaccination. We proposed that this knowledge would lead to greater insight into protective mechanisms against the disease, which, in turn, would inform vaccine development.

We applied a systems biology approach to analyze unique prospective samples, and showed that in healthy infants who displayed different patterns of immune responses to the BCG vaccine, distinct mechanisms may underlie susceptibility to future TB disease, suggesting that the same intervention may not benefit all.

## Methods

### Study participants, blood collection and follow-up

**Fig. 1:**
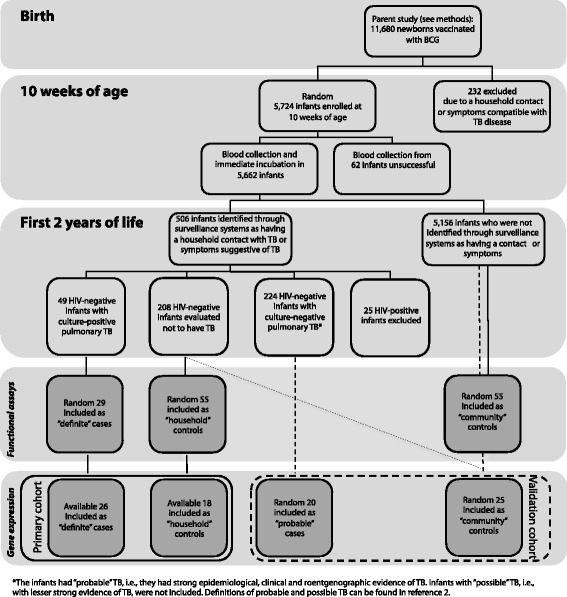
Cohort of infants vaccinated with BCG at birth. At 10 weeks of age, blood was collected from HIV-negative, HIV-unexposed infants with no active or chronic illnesses (including suspected TB), and with no household exposure to an adult who was coughing, or who had TB disease. Infants were then followed for 2 years. Community-wide surveillance systems identified all children exposed to adults with TB, or children with suspected TB disease. Among these children, “definite” TB cases were defined by presence of clinical signs and symptoms of lung disease plus a sputum (induced, or early morning gastric aspirate) culture positive for *M. tuberculosis*, while “probable” TB cases were defined by absence of a positive culture in the presence of strong epidemiological, clinical and chest roentgenographic evidence of TB disease. Two groups of controls were identified: “household” controls were exposed to an adult in the household with TB but were found not to have TB, whereas “community” controls were infants who were either investigated for TB and found not to have disease, or infants chosen at random from the rest of the cohort. For functional assays, up to 29 definite cases and 110 controls (household controls, n = 55, and community controls, n = 55) were included in different analyses. Primary analysis of transcriptional profiling was restricted to those cases and controls included in functional assay analysis for whom PBMC were available

We enrolled infants at the South African Tuberculosis Vaccine Initiative (SATVI) field site, near Cape Town, South Africa. This area has a high TB disease incidence in children < 2 years of age (>1,000/100,000/year). This study was nested within a randomized controlled trial (RCT) [2], which aimed to determine whether intradermal or percutaneous delivery of Japanese BCG at birth resulted in equivalent protection against TB. For our correlates of risk study, we enrolled 5,724 infants, randomly, from the 11,680 infants enrolled in the RCT, when they were 10 weeks of age, as previously described [3] (Fig. 1). The following were exclusion criteria: mothers known to be HIV-infected, BCG not received within 24 h of birth, significant perinatal complications, any acute or chronic disease in the infant, clinically apparent anemia in the infant, and household contact of the infant with any person with TB, or any person who was coughing.

At 10 weeks of age, blood was collected. Infants were then followed for 2 years. Community-wide surveillance systems, described for the parent study [2], identified all children exposed to adults with TB, or children with suspected TB disease. These children were admitted to a dedicated case verification ward for evaluation [2]. Investigations included clinical and epidemiologic evaluation, two induced sputa and gastric aspirates for mycobacterial culture, chest roentgenography and a tuberculin skin test [2]. Cases included in the current study were either culture positive for *Mycobacterium tuberculosis* (primary analysis), or were not culture positive but there was strong clinical, roentgenographic and epidemiological evidence of TB disease (validation analysis). Infants who were evaluated in our case verification ward and were found not to have TB were included as controls (primary analysis). Some of the control infants included in the validation analysis lived in the same endemic area but did not meet the criteria for TB investigation (community controls).

Human participation was according to the US Department of Health and Human Services and good clinical practice guidelines. This included protocol approval by the University of Cape Town Research Ethics Committee (reference 016/2001), and written informed consent from the parent or legal guardians.

### Gene expression analysis

#### PBMC isolation, storage, and *in vitro* incubation for RNA isolation and amplification

Peripheral blood mononuclear cells (PBMC) were isolated from peripheral blood of 10-week-old infants by density gradient centrifugation and cryopreserved. Later, PBMC were thawed, rested, and 10^6^ cells were incubated in culture with either BCG (Danish strain, Statens Serum Institut, reconstituted as previously described [4]) at a multiplicity of infection (MOI) of 0.18, or with medium alone. PBMC were incubated for 12 h at 37 °C and 5 % CO_2_. Following the incubation period, cells were harvested and RNA extracted using the QIAamp RNA Blood Mini Kit (Qiagen), according to the manufacturer’s instructions. A median of 0.92 μg (range 0.24–1.56 μg) RNA was obtained from 10^6^ PBMC. Extracted RNA was cryopreserved at −80 °C. Later, RNA was thawed and a median 339 ng (range 88–595 ng) amplified using the Illumina RNA Amplification Kit (Ambion), as previously described [4]. The median RNA yield after amplification was 15.3 μg (range 2.35–32.8 μg).

#### DNA microarray

Amplified RNA (750 ng per array) was hybridized to the Illumina HumanRef-8 Expression BeadChip (version 2), according to the manufacturer’s instructions. Arrays were scanned with an Illumina bead array reader confocal scanner. Array data is now housed at the gene expression database repository Gene Expression Omnibus (GEO) submission number GSE20716 [5].

#### Microarray data analysis

Raw Illumina probe data were exported from BeadStudio and screened for quality. Scanned array images were visually inspected for artifacts or mishandling. Diagnostic plots, including density plots, box plots and heatmaps of between-array distances, were used to assess hybridization quality within the whole data set. One array failed quality screening. Pre-processing and statistical analysis were conducted using the R statistical language and various software packages from Bioconductor (www.r-project.org, version 3.1.2) [6]. Quantile normalization was applied, followed by a log2 transformation. Bioconductor’s genefilter package was used to filter out genes with low expression (genes retained with expression values greater than 100 or 200 units in at least two samples) and insufficient variation in expression as measured by the interquartile range (IQR; genes retained with IQR higher than 0.2, 0.3 and 0.4) across all samples tested.

##### Unsupervised clustering and class assignment analysis

Unsupervised cluster analysis was performed, using R’s cluster package, which utilizes hierarchical clustering. This hierarchical clustering approach has been successfully used to identify clinically relevant cancer sub-types in several studies [7, 8]. PBMC were incubated with BCG or with media alone for 12 h prior to measuring gene expression. Expression from the unstimulated condition was subtracted from that in the BCG-stimulated condition; unsupervised clustering was used to identify potential sub-groups of infants. Using the genefilter package with four different filtering criteria, we selected different numbers of probes to assess the stability of clustering: probes retained with expression values greater than 100 in at least two samples and IQR range in expression across samples > 0.2 (9,878 probes), probes retained with expression values greater than 200 in at least two samples and IQR range in expression across samples > 0.2 (7,902 probes), probes retained with expression values greater than 200 in at least two samples and IQR range in expression across samples > 0.3 (5,306 probes), and probes retained with expression values greater than 200 in at least two samples and IQR range in expression across samples > 0.4 (3,077 probes). The selected probes were used as input to test the stability and robustness of the clustering process. Two major unchanged cluster groups of 18 (cluster 1) and 15 (cluster 2) core infants were observed across the four different pre-clustering conditions. The filtering was performed before the unstimulated condition was subtracted from that in the BCG-stimulated condition. There were 11 infants with an unstable cluster membership as they switched membership from cluster 1 to cluster 2 dependent upon input gene number (Additional file 1: Figure S1). The 33 donor samples were then used to train a two-class binary classifier using *pamr*, a class prediction tool implemented in R, to perform a class assignment for the 11 unstable infants. The 11 remaining donors were classified, six in cluster 1 group and five in cluster 2 group. Similar class assignment was also obtained using a *k-*Nearest Neighbors classification algorithm, with *k* = 3.

##### Identification of differentially expressed genes

Bioconductor’s limma package was used to identify differentially expressed genes (https://bioconductor.org/packages/release/bioc/html/limma.html). This approach estimates the fold change (FC) between predefined groups by fitting a linear model and using an empirical Bayes method to moderate standard errors of the estimated log-fold changes for expression values from each gene. The *p* values from the resulting comparison were adjusted for multiple testing according to the method of Benjamini and Hochberg [9]. This method controls the false discovery rate (FDR), which was set to 0.05 (5 % FDR). Limma analysis was then used to identify genes significantly differentially expressed between cluster 1 and cluster 2 infants (5 % FDR). We also used limma to identify genes significantly differentially expressed in a one-versus-all analysis comparing each group (cluster 1 cases, cluster 1 controls, cluster 2 cases and cluster 2 controls) with a pool of the three other groups.

##### Pathway analysis

Pathway analysis was performed using Gene Set Enrichment Analysis (GSEA), a non-parametric annotation-driven statistical analysis method [10]. Analysis of groups of genes encompassed into distinct signal transduction or transcriptional pathways ultimately yields greater insight into biological processes than simple exploration of lists of differentially expressed genes [11, 12]. The basic principle of GSEA is to use a list of genes ranked by FC to determine whether a known biological pathway or sets of individual genes are significantly enriched in one phenotype when compared to another. To evaluate this degree of enrichment the GSEA method calculates an enrichment score (ES) based on the Kolmogorov–Smirnov statistic. The statistical significance of a gene set’s ES is estimated by an empirical phenotype-based permutation test procedure. To account for multiple hypotheses testing, GSEA normalizes the ES for each gene set to account for variation in set sizes and calculates a FDR corresponding to each normalized ES.

We systematically tested gene sets from the Molecular Signatures Database (MSigDB; http://www.broad.mit.edu/gsea/msigdb) C2 collection (c2.cp.v3.0.symbols.gmt), collected from various online canonical pathways databases, including KEGG, BioCarta, Reactome and NetPath, to which we added a collection of immune-related gene sets described in Chaussabel *et al*. [13].

An over-representation test was performed using Fisher’s exact test. Statistical significance of *p* < 0.05 (5 % FDR) was used to identify gene signature enrichment from a list of genes selected as differentially expressed between two groups of samples.

### Whole blood intracellular cytokine assay

At 10 weeks of age, heparinized blood was collected from all infants. One milliliter was immediately incubated with BCG (1.2 × 10^6^ organisms/mL), as previously described [3]. Medium alone served as negative control, while staphylococcal enterotoxin B (SEB; Sigma-Aldrich, 10 μg/mL) was used as a positive control. The co-stimulatory antibodies anti-CD28 and anti-CD49d (BD Biosciences, 1 μg/mL each) were added to all conditions, as this results in enhancement of specific T cell responses. Blood was incubated for 7 h at 37 °C. At this time, plasma supernatants were harvested and cryopreserved at −80 °C. Brefeldin A was then added, followed by incubation for an additional 5 h. Cells were harvested, fixed and cryopreserved, as previously described. Later, cells were thawed, permeabilized, and stained for T cell surface markers and for intracellular TNF-α, IFN-γ and IL-2, as previously described. Frequency of CD4+ and CD8+ T cells expressing these cytokines was detected by a LSRII flow cytometer (BD Biosciences), using FACSDiva 6.1 software.

### Soluble cytokine/chemokine measurement

Plasma was thawed once and levels of 29 cytokines/chemokines determined with the human cytokine LINCO*plex* 29-bead array assay kit (LINCO Research, Millipore). The following cytokines/chemokines were measured: interleukin-1 alpha (IL-1α), IL-1β, IL-1RA, IL-2, IL-4, IL-5, IL-6, IL-7, IL-8, IL-10, IL-12p40, IL-12p70, IL-13, IL-15, IL-17, soluble CD40 ligand (sCD40L), epidermal growth factor (EGF), eotaxin, fractalkine, granulocyte-colony stimulating factor (G-CSF), granulocyte-macrophage colony-stimulating factor (GM-CSF), interferon-gamma (IFN-γ), 10 kDa interferon gamma-induced protein (IP-10), monocyte chemoattractant protein-1 (MCP-1), macrophage inflammatory protein-1 alpha (MIP-1α), MIP-1β, transforming growth factor-alpha (TGF-α), tumor necrosis factor-alpha (TNF-α) and vascular endothelial growth factor (VEGF). The manufacturer’s instructions were followed. Fluorescence was detected using a Luminex 100 IS machine (xMAP Technology, Luminex Corporation), using Luminex software.

### Lymphocyte proliferation assay

Cryopreserved PBMC were thawed in culture medium (12.5 % v/v AB+ serum in RPMI) containing 10 μg/mL DNase (Sigma-Aldrich). After washes, cells were stained with 1 μg/mL Oregon Green (Molecular Probes) and rested overnight at 37 °C in 5 % CO_2_. PBMC (2 × 10^5^/well) in 200 μL culture medium (12.5 % v/v AB serum in RPMI) were incubated with BCG at a MOI of 0.01 for 6 days, at 37 °C in 5 % CO_2_. SEB (0.5 μg/mL) served as positive control, while medium only served as negative control. Cells were harvested with 2 mM EDTA (Sigma-Aldrich) in PBS and stained with 1 μg/mL LIVE/DEAD Fixable Violet Dead Cell Stain (Invitrogen). Cells were fixed with FACS Lysing Solution (BD Biosciences) and cryopreserved. Later, cells were thawed, washed, and stained for 1 h at 4 °C with anti-CD3 Qdot605 (clone UCHT1) and anti-CD8 Cy5.5PerCP (SK1), both from BD Biosciences. Cells were acquired as described above. Anti-mouse kappa beads (BD Biosciences), stained with the respective fluorescent-labeled antibody, were used to configure compensation settings.

### Cytotoxic marker assay

Thawed PBMC (2 × 10^5^ cells/well) in 200 μL culture medium were incubated with BCG at a MOI of 0.1, at 37 °C in 5 % CO_2_. Controls included SEB (0.05 μg/mL) or medium alone. After 72 h, cells were harvested, stained with the violet viability dye, fixed and cryopreserved, as described above. Later, cryopreserved, fixed cells were thawed, washed, permeabilized with Perm/Wash solution (BD Biosciences) and stained with the following antibodies for 1 h at 4 °C: anti-CD3 Qdot605 (clone UCHT1), anti-CD8 Cy5.5PerCP (SK1), anti-granzyme B Alexa 700 (GB11), anti-perforin FITC (δG9; all from BD Biosciences) and anti-granulysin PE (eBioDH2, eBioscience), acquired as described above.

### Quantification of myeloid and lymphoid cell populations in PBMC

Cryopreserved PBMC from case and control infants were thawed and washed, and immediately stained. Cells were stained for 30 min at 4 °C with the following antibodies: CD45 FITC (clone 2D1), CD19 PE (clone HIB19), CD3 APC (clone UCHT1; all from BD Biosciences), CD11c PerCP-cy5.5 (clone Bu15), HLA-DR Alexa Fluor 700 (clone L243; both from BioLegend), CD14 Qdot605 (clone Tuk4) and LIVE/DEAD Fixable Violet Dead Cell Stain (both from Invitrogen). Samples were acquired on a LSRII flow cytometer and the analysis was performed using FlowJo (version 9.3.1, Tree Star). The frequencies of CD14+ monocytes, HLA-DR+ CD11c + mDC, CD3+ and CD19+ lymphocytes were expressed as relative proportions of live CD45+ leukocytes.

### Analysis of non-expression data

Flow cytometric data were analyzed using FlowJo (version 8.8.4, Tree Star). Whole blood intracellular cytokine assay was analyzed as previously described [3]. For the proliferation assay, only infants who had a frequency of Oregon Green^low^ CD8− T cells, following SEB incubation, that was greater than the median frequency plus 3 median absolute deviations (MAD) of the negative control, were included in the analysis. For the cytotoxic marker assay, results from infants who had a frequency of granzyme B-expressing CD8+ or CD8− T cells, following SEB incubation, that was greater than the median frequency plus 3 MAD of the negative control, were included.

Cytokine data were imported directly into Microsoft Excel for analysis, from the Luminex software. Duplicate standard curves were generated for each cytokine from the standards; these curves were used to calculate cytokine levels. Since many analytes were already secreted at broadly variable levels in unstimulated samples, we did not subtract values from unstimulated samples, and we report concentrations upon stimulation with BCG, unless otherwise indicated.

For univariate assessment of differences between infant groups, a Mann–Whitney U test was performed, using Prism (GraphPad Software, Inc.).

## Results

### Participants

Our systems biology approach combined unbiased transcriptional profiling with hypothesis-driven characterization of innate and T cell responses thought to be important for TB control. Blood was collected, processed and stored at 10 weeks of age from a random 5,726 healthy infants, who belonged to a parent cohort of infants routinely vaccinated with BCG at birth [2] (refer to Fig. 1 for detail). During 2 years of follow-up, infants who developed pulmonary TB were identified (cases), as well as those who did not develop TB disease (controls) [2].

For functional assays, up to 29 definite cases and 110 controls (household controls, n = 55, and community controls, n = 55) were included in different analyses.

Primary analysis of transcriptional profiling was restricted to those cases and controls included in functional assay analysis for whom PBMC were available. Two groups of cases were identified: for primary analysis, infants with culture positive pulmonary TB (“definite” cases, n = 26 were included in analysis), and for validation analysis, infants with strong epidemiological, clinical and roentgenographic evidence of TB disease, without a positive culture—the latter is the most common clinical presentation of childhood TB (“probable” cases, n = 20). Two groups of controls were identified: for primary analysis, infants with household exposure to an adult with TB disease and found not to have TB (“household” controls, n = 18), and for validation analysis, infants similarly defined plus infants who were either investigated for TB and found not to have disease, or infants chosen at random from the rest of the cohort (“community” controls, n = 25). The definition of primary and validation cohorts differed because of limited sample sizes.

### Correlates of prospective risk of TB disease could not be identified

For transcriptional profiling, PBMC isolated at 10 weeks of age from cases and controls were incubated with BCG or medium alone for 12 h. RNA was extracted and analyzed by DNA microarrays, as previously described [4]. No difference could be shown between cases and controls when gene expression from PBMC incubated with medium only was subtracted from that in PBMC incubated with BCG (Additional file 1: Figure S2, Additional file 2: Table S1; two genes were differentially expressed with a nominal *p* value less than 0.05); neither could differences be found when BCG-induced gene expression alone was examined independently (Additional file 2: Table S1; 25 genes were different with a nominal *p* value less than 0.05), nor when gene expression in medium alone was examined independently (Additional file 2: Table S1; 32 genes were different with a nominal *p* value less than 0.05).

Multiple additional assays were completed to compare cases and controls, focusing on immune activation thought to be important for control of TB. In whole blood incubated with BCG, there was no difference in frequency of specific Th1 cells [3], nor release of pro- and anti-inflammatory mediators (Additional file 1: Figure S3). In PBMC incubated with BCG, there was no difference in proliferation of T cells (Additional file 1: Figure S4), nor T cell expression of cytotoxic molecules (Additional file 1: Figure S5).

Finally, we tested whether frequencies of myeloid and lymphoid cellular subsets in PBMC differed, because of multiple reports of associations between ratios of these cells and various infectious diseases and vaccination outcomes [14–17]. No difference was shown (Additional file 1: Figure S6).

### Two distinct patterns of gene expression when cases and controls were examined together

**Fig. 2:**
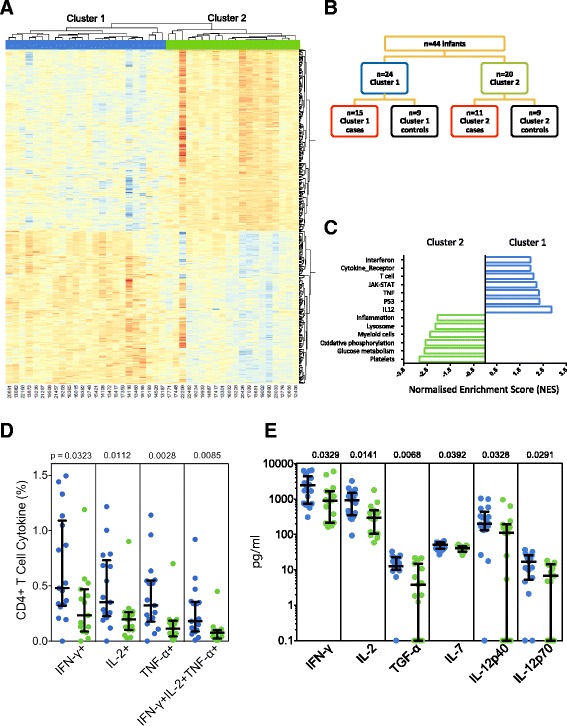
Identification of two clusters of gene expression in infants. Samples from the primary cohort of definite cases and household contacts were examined together. **a** Unsupervised clustering analysis was performed as described in “Methods”, resulting in two major clusters of infants. The heatmap shows results of supervised hierarchical clustering analysis (Pearson correlation), using genes differentially expressed between infants belonging to the two clusters (Additional file 1: Figures S1 and S2); the normalized probe intensity obtained from PBMC stimulated with media only was subtracted from the normalized probe intensity obtained from PBMC stimulated with BCG for 12 h. **b** Representation of cases and controls among the two clusters. **c** Gene expression within biological pathways, identified by GSEA, that differed between cluster 1 (*blue*) and cluster 2 (*green*). Representative GSEA pathways were ranked by FDR *q* value (and *p* value < 0.05). **d** Frequencies of BCG-specific CD4+ T cells among infants from cluster 1 (*blue circles*) or cluster 2 (*green circles*). Antigen-specific cells were identified by cytokine expression following incubation of whole blood with BCG for 12 h. *Bars* depict medians and IQR; the Mann–Whitney U test was used to assess differences. **e** Quantification of molecules released in whole blood after a 7-h incubation with BCG. BCG-specific levels were calculated by subtracting levels in plasma from whole blood incubated with costimulatory antibodies alone from those in BCG-stimulated blood. *Bars* depict medians and IQR; the Mann–Whitney U test was used to assess differences

We hypothesized that divergent host responses following BCG vaccination were responsible for the inability to show correlates of risk of TB disease on a population basis. We therefore examined gene expression of definite TB cases and household contacts in the primary cohort. Expression data from PBMC incubated with media alone was subtracted from that of PBMC incubated with BCG for 12 h. Unsupervised analysis revealed two major clusters: one containing 24 infants—cluster 1—and one containing 20 infants—cluster 2 (Fig. 2a, b). Clustering was not associated with being a case or a control (Fig. 2b, Additional file 1: Figure S2), and was stable, as altering input gene number did not impact cluster membership for the majority of infants (Additional file 1: Figure S1). We found no clinical variable that differed between the two clusters, including vaccination route, weeks of gestation, gender, ethnicity and birth weight (not shown); there were also no experimental variables that differed, including RNA quantity and quality (not shown); and there was no significant difference between the time to TB diagnosis in cluster 1 and cluster 2 case infants (data not shown).

Overall, 461 genes were differentially expressed between the two clusters (FC > 1.3 and FDR < 5 %; Fig. 2, Additional file 3: Table S2). Differential expression of these genes could also identify two clusters of infants in the validation cohort of probable TB cases and community controls (Additional file 1: Figure S7A, Additional file 4: Table S3).

GSEA showed pathways that were unique to each cluster. The primary cohort demonstrated enrichment (FDR < 5 %) of gene sets associated with interferons, T cells, cytokines and JAK-STAT signaling in cluster 1, while gene sets associated with inflammation distinct from those in cluster 1, plus gene sets of myeloid cells, were enriched in cluster 2 (Fig. 2c, Additional file 5: Table S4). Again, the validation cohort confirmed these findings (Additional file 1: Figure S7B).

Next, we assessed whether results from hypothesis-driven cellular and soluble marker assays used to interrogate differences between cases and controls (above) would align with the distinct gene expression pathway findings. This was the case, as cluster 1 infants showed a higher frequency of BCG-specific Th1 cells and greater Th1-polarizing cytokine release, compared with cluster 2 (Fig. 2d, e).

Collectively, these results suggest that infants routinely vaccinated with BCG vaccination at birth display two distinct patterns of immune activation.

### Clustering is independent of BCG stimulation *ex vivo*

Next, we wished to learn whether clustering of gene expression was dependent on antigenic stimulation. Using expression data from PBMC from the primary cohort incubated with media alone, we showed that 670 genes were differentially expressed between cluster 1 and 2 infants in the absence of antigen stimulation (Limma analysis, FDR < 0.05 and FC > 1.3; Additional file 1: Figure S8). Using these 670 genes, the majority of individual participants fell into the same clusters as reported above, generated when expression data from PBMC incubated from media alone was subtracted from that of PBMC incubated with BCG, with only three exceptions (Additional file 1: Figure S8). Similarly, in the validation cohort, 3,221 transcripts were differentially expressed (FDR < 0.05 and FC > 1.3) between cluster 1 and cluster 2; clustering was found to be independent of BCG antigen stimulation (data not shown).

**Fig. 3:**
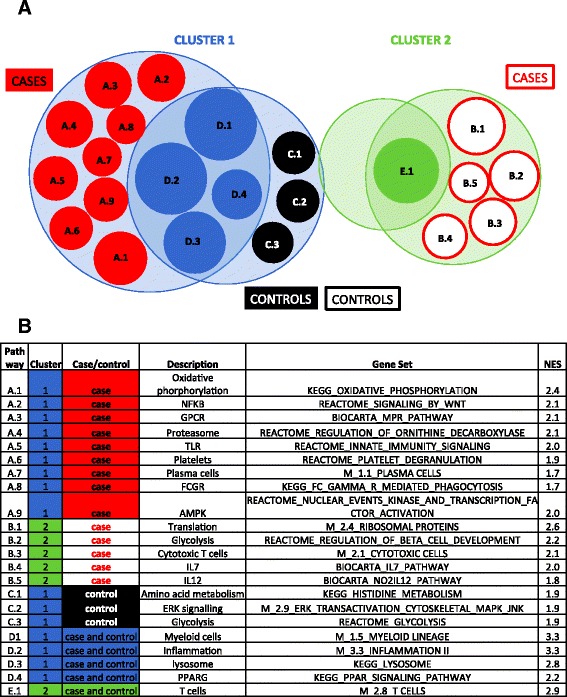
Pathway analysis, using gene expression data from unstimulated PBMC, in the two clusters of infants. Samples from the primary cohort were analyzed. A one group versus all other groups analysis approach was used to identify genes differentially expressed in each of the four groups, when compared to a pool of the three other groups (Additional file 4: Table S3). Then, genes were ranked according to the moderated *t* test and GSEA used to identify pathways enriched in each of the four groups. Finally, pathways uniquely and commonly expressed within each group were identified, using a FDR adjusted *p* value cutoff of < 0.05. The Venn diagram (**a**) shows both shared and unique gene sets, which are identified in (**b**). Gene sets are sized by normalized enrichment score (NES)

Pathway analysis (GSEA), using results from the unstimulated samples, in the primary cohort showed that gene sets representing myeloid cells and anti-inflammatory responses were prominent in cluster 1 (Fig. 3b). In contrast, pathways commonly expressed in cluster 2 were associated with T cells (Fig. 3b). Again, similar clustering was also shown in unstimulated samples from the validation cohort (data not shown).

Taken together, these findings suggest that after BCG vaccination of newborns, underlying differences in the two clusters exist that are independent of specific antigen stimulation, and which may determine outcome of antigen exposure.

### Distinct cellular and metabolic pathways among cases, and among controls, from each of the two clusters

Next, to learn about the possible association of clustering with risk of TB disease, we used pathway analysis (GSEA) of gene expression from unstimulated samples in the primary cohort to identify pathways uniquely expressed in each of four groups: a set of cases and controls from each cluster (Fig. 3, Additional file 5: Table S4). Pathways associated with oxidative phosphorylation and activated MAP kinase were uniquely enriched in cluster 1 cases, while those of amino acid metabolism and glycolysis were enriched in cluster 1 controls (Fig. 3, Additional file 6: Table S5). Pathways associated with T cell activation, including glucose metabolism, IL-7, IL-12 and ribosomal protein translation [18, 19], were the most prominent in cluster 2 cases (Fig. 3, Additional file 6: Table S5). We could not identify pathways unique to cluster 2 controls (Fig. 3, Additional file 6: Table S5). Similar patterns were found in the validation cohort for cluster 1 cases and controls (Additional file 1: Figure S7, Additional file 7: Table S6), but no pathway enrichment was shown in cluster 2 infants of the validation cohort.

It should be noted that in unstimulated PBMC for both the primary and validation cohorts, myeloid and inflammatory gene signatures decreased upon BCG antigen stimulation. This decline was greater in extent in cluster 1, compared with cluster 2 (Additional file 1: Figure S9); therefore, an antigen-specific decrease in myeloid and inflammatory signatures is associated with a rise in Th1 cytokine release.

Together, these findings confirm different host responses in different groups of infants following BCG vaccination, and suggest that distinct mechanisms could underlie TB disease risk in different groups of infants.

### High monocyte to T cell ratios and higher specific T cell responses may be associated with risk of TB disease, in one cluster

**Fig. 4:**
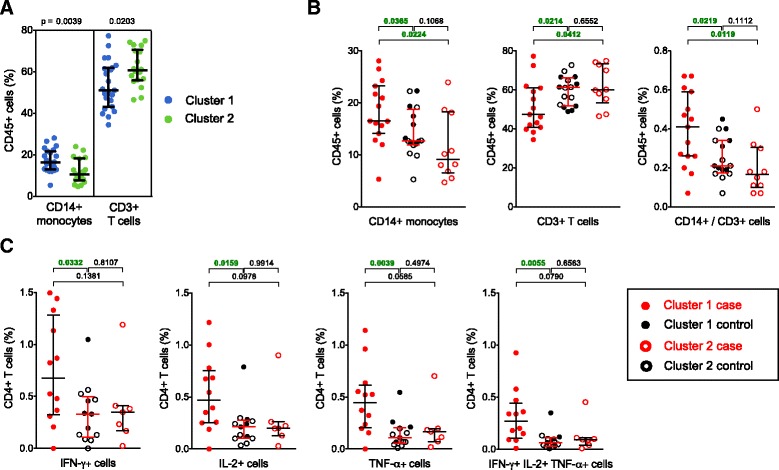
Cellular phenotype and function in case and control infants from cluster 1 and cluster 2. Samples from the primary cohort were analyzed. **a** Frequencies of monocytes and T cells, measured by flow cytometry (Additional file 1: Figure S6) in unstimulated PBMC from infants in cluster 1 (*blue circles*, n = 23) and cluster 2 (*green circles*, n = 18). **b** Comparison of the cellular phenotype of definite TB case infants from cluster 1 (*red closed circles*, n = 15) and from cluster 2 (*red open circles*, n = 10) with that of pooled household controls (*black closed circles* for cluster 1, n = 8, and *black open circles* for cluster 2, n = 8). CD14+ monocytes, CD3+ T cells and the ratio of monocytes divided by lymphocytes are shown. **c** Frequencies of CD4+ T cells expressing either IFN-γ, IL-2 or TNF-α, or all three cytokines together, measured by intracellular cytokine staining and flow cytometry, following whole blood stimulation with BCG for 12 h. For this analysis, definite TB case infants from cluster 1 (*red closed circles*, n = 12) and from cluster 2 (*red open circles*, n = 7) were compared to pooled household controls (*black closed circles* for cluster 1, n = 5, and *black open circles* for cluster 2, n = 8). *Bars* depict medians and IQR; the Mann–Whitney U test was used to assess differences in all analyses

To explore whether observed differences in gene expression were associated with relative abundance of cellular subsets within PBMC, we quantified frequencies of myeloid cells (monocytes and myeloid dendritic cells) and lymphoid cells (T and B cells) with flow cytometry (Additional file 1: Figure S6). Cluster 1 infants had higher frequencies of CD14+ monocytes and lower frequencies of CD3+ T cells in PBMC, compared with cluster 2 infants (Fig. 4a), which aligned with relatively higher myeloid pathway activation seen in cluster 1 (Fig. 3).

Since our sample sizes were small, we pooled the controls from each cluster for further flow cytometric analyses. This was justified because only small differences in gene expression between the two groups of controls were shown—only seven genes were differentially expressed (Additional file 6: Table S5); in contrast, the two groups of cases showed 646 differentially expressed genes (Additional file 7: Table S6). Cases in cluster 1 displayed higher monocyte to T cell ratios, compared with the pool of controls and with cluster 2 cases (Fig. 4b). No differences were observed for the other cell subsets, including B cells (data not shown).

We then showed that cluster 1 cases had higher frequencies of BCG-specific CD4+ T cells producing type 1 cytokines (Fig. 4c), and that BCG induced greater upregulation of granulysin and perforin (Additional file 1: Figure S10A), compared with pooled controls. CD8+ and γδ TCR+ T cell responses to BCG, as well as BCG-specific CD4+ and CD8+ T cell proliferation, did not differ between groups (Additional file 1: Figure S10B and data not shown).

These results suggest that in a major cluster of BCG vaccinated infants, the presence of high frequencies of monocytes, or of a specific type 1 cellular immune response, both regarded as important for control of mycobacteria, might be associated with higher risk of developing TB disease.

### M2 alternatively activated and M1 classically activated monocytes in different clusters of cases

**Fig. 5:**
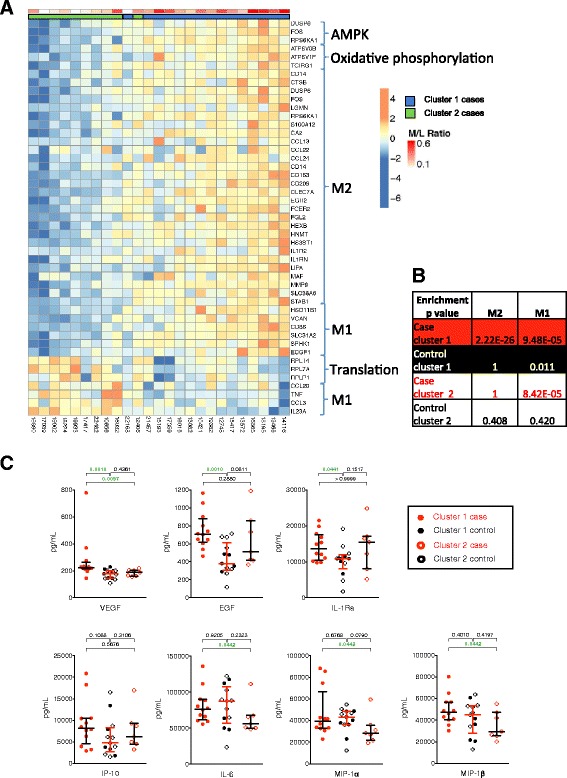
Evidence of differential M1 and M2 monocyte activation in cases and controls from different clusters. Samples from the primary cohort were analyzed. **a** Heatmap showing genes associated with AMPK, oxidative phosphorylation, translation, M1 monocytes and M2 monocytes, which were differentially expressed between cluster 1 cases and the three other groups, or cluster 2 cases and the three other groups. Cases from clusters 1 and 2 are shown. To build the heatmap, the infants were first ranked by increasing expression intensity for each gene. Then, the mean-rank, across the set of genes, for each infant was used to order infants from the lowest to the highest mean-rank. A Spearman correlation was used to assess the significance (*p* value < 0.0008) of the association between the ordering of the infants and the monocyte to T cell ratio. **b** The *p* value table from an over-representation test performed using Fisher’s exact test to identify M1 (*right column*) and M2 (*left column*) gene signature enrichment amongst up-regulated in each of the four groups of infants. (**c**) Concentrations of pro- and anti-inflammatory molecules were measured by Luminex in plasma from whole blood stimulated with BCG for 7 h. *Bars* depict medians and IQR; the Mann–Whitney test was used to assess differences in all analyses

Next, we explored gene expression pathways enriched in different clusters of case infants in greater detail. Genes in the M1 or M2 monocyte pathways showed an association with ratio of myeloid to lymphoid cellular frequency in case infants (Fig. 5a). Pathways in cluster 1 cases included oxidative phosphorylation, which is the mechanism that activated anti-inflammatory monocytes, or M2, use to gain energy for function and survival [18]. A detailed analysis revealed enrichment for expression of genes characteristic of both M1—classically activated monocytes—and M2 in cluster 1 cases, compared with the other group of cases and the controls (Fig. 5b). Cluster 2 cases showed enrichment of a sub-set of M1 genes (Fig. 5b). Cluster 1 controls showed modest enrichment of M1 genes, possibly related to higher frequencies of monocytes observed in cluster 1 infants (Figs. 4a, 5b).

We evaluated whether whole blood production of cytokines associated with M1 and M2 would follow gene expression patterns. Production of EGF in unstimulated blood, and of EGF, VGEF and IL-1Ra—all associated with M2 macrophages [20]—in BCG-stimulated blood modestly confirmed gene expression patterns (Fig. 5c). Conversely, IFN-γ-driven expression of IP-10, typically from M1 monocytes, was similar across groups, again supporting gene expression results (Fig. 5c). Pro-inflammatory IL-6, MIP-1α and MIP-1β, typically from M1 monocytes, were slightly elevated in cluster 1 cases, compared with cluster 2 cases (Fig. 5c).

These findings suggest that monocyte phenotype may additionally contribute to differences in immunogenicity in the two infant clusters.

## Discussion

Our primary aim was to determine correlates of risk of TB disease following BCG vaccination of newborns. Despite combining unbiased and hypothesis-driven approaches, no such correlates could be identified. This may be due to the observation that, on a population level, infants vaccinated with BCG at birth demonstrated two distinct clusters of immune response, as measured by global transcriptional response to *in vitro* stimulation with BCG. Within these clusters, infants at risk of TB disease showed different gene expression, suggesting that unique correlates of risk may not be found within clusters. Our sample size was not adequate to do this analysis definitively, but was sufficient for learning about distinct immune responses within clusters. Immediate implications of this observation are that future studies aimed at defining correlates of risk of, or of protection against, TB disease should plan for adequate sample sizes to address heterogeneity, and that analysis strategies should account for heterogeneity.

We observed that infants with the highest or lowest ratios of monocytes to T cells were at risk of developing TB disease. We have also used full differential blood count data available from study cohorts in Durban, South Africa, to show that both high and low monocyte to lymphocyte ratios were associated with TB disease risk in HIV-exposed infants, post-partum mothers and adults initiating anti-retroviral therapy [14–16]. It has previously been shown that hematopoietic stem cells can directly respond to IFN-γ mobilizing myeloid cells in response to mycobacterial infections [21]. In the absence of *M. tuberculosis* infection, it is therefore possible that persistence of live attenuated BCG from vaccination at birth may have driven myeloid cell expansion seen in case infants with higher monocyte to T cell ratios. We cannot exclude that host genetic determinants, other infections, other immunizations, or environmental determinants, such as nutrition, might also be important; we did not find influence of other infections or environmental co-variates in our infants. Future studies will address these variables in a systematic fashion.

We further showed that case infants with higher monocyte to T cell ratios have a transcriptional profile associated with an alternatively activated macrophage phenotype (M2), modestly confirmed by soluble marker measurement data. The M1/M2 pathways used in this study included those defined but not yet included in the MSigDB database [22], and pathways associated with the development of M2 phenotype, such as activated MAP kinase signaling and platelets [18, 23]. The latter two functions have been shown to drive monocyte differentiation into giant cells, which can suppress the mycobacteria-specific immune response [18, 23]. We have previously shown that healthy BCG vaccinated infants have M1 macrophage phenotype [4, 24], suggesting that the usual host response to BCG immunization involves classically activated monocytes (M1 activation was seen in all cases and controls in the current study, with M2 in addition in the case group referred to above). Typically, M1 macrophages are associated with killing of mycobacteria, whereas M2 macrophages are associated with tissue repair and bacterial persistence [25, 26]. Therefore, activation of M2 cells may be involved in pathogenesis of risk of TB disease.

It is widely accepted that T cells are critical for control of *M. tuberculosis* infection, and to prevent progression to active TB disease [27–29]. The importance of CD4+ T cells is evident in persons with HIV-1-associated depletion of CD4+ T cell numbers and function; while not on antiretroviral therapy, these individuals are at > tenfold greater risk of progression from infection to disease [30]. Humans with mutations of genes in the Th1 response axis are highly susceptible to mycobacterial disease [31]. As a consequence, IFN-γ expression has been considered as a correlate of induced immunity, or “vaccine take”, in clinical trials of novel TB vaccines. However, we have recently shown that vaccine-induced antigen-specific Th1 responses did not correlate with risk of TB disease, following BCG vaccination of newborns: frequencies of BCG-specific IFN-γ-expressing, polyfunctional (co-expressing IFN-γ, TNF-α and IL-2) or IL-17-expressing CD4+ T cells, as well as IFN-γ-expressing CD8+ T cells, did not correlate with risk [3]. Surprisingly, in the current study higher frequencies of BCG-specific Th1 and polyfunctional cells were detected in a cluster of infants at risk of TB disease; the same infants also had M2 monocyte activation. Together, these findings suggest that a strong Th1 response in a context of heightened alternative activation of monocytes may be detrimental to the host.

## Conclusions

Our findings suggest that distinct patterns of host responses to *Mycobacterium bovis* BCG are present in infants vaccinated with BCG. Vaccines for intracellular pathogens are designed to boost Th1 immune responses, which may be an effective strategy for immune response cluster 2 infants, but may not be effective for protection of cluster 1 infants. It is tempting to hypothesize that partial efficacy observed with candidate vaccines for TB, HIV and malaria [32–34] can be ascribed to differential host responses within populations. Indeed, we have shown that the monocyte/lymphocyte ratio impacts on the efficacy of the malaria vaccine RTS,S, which protects children with lower ratio (similar to immune responses in our cluster 2), but not higher ratio (similar to immune responses in our cluster 1) in a phase II efficacy trial in Kilifi, Kenya [17].

## Additional files

Additional file 1: Figure S1. Stability of the two major gene expression clusters. **Figure S2.** Gene expression in cases and controls. **Figure S3.** BCG-specific induction of soluble cytokine/chemokine production in cases and controls. **Figure S4.** Frequencies of proliferating BCG-specific T cells in cases and controls. **Figure S5.** Frequencies of specific T cells expressing cytotoxic molecules in cases and controls. **Figure S6.** Frequencies of myeloid and lymphoid cellular subsets in cryopreserved PBMC in cases and controls. **Figure S7.** Gene expression in the validation cohort of probable TB cases and community controls. **Figure S8.** Clustering of gene expression in unstimulated samples. **Figure S9.** *In vitro* modulation of gene expression by BCG. **Figure S10.** Frequencies of antigen-specific CD4 and CD8 T cells expressing cytotoxic molecules, and of proliferating BCG-specific T cells, in cases of each cluster, and in controls. (PDF 2443 kb) Additional file 2: Table S1. Number of genes differentially expressed between cases and controls from the primary cohort. Column B shows this analysis when all infants were included, whereas the other two columns show analysis with cluster 1 and cluster 2 infants included, respectively. A non-FDR adjusted *p* value cutoff of 0.05 and a fold change absolute value of 1.3 was used to define differential expression. (XLSX 11 kb) Additional file 3: Table S2. Genes differentially expressed between cluster 1 and cluster 2 infants from the primary cohort. Gene expression was calculated by subtracting expression in PBMC incubated with medium alone from expression in PBMC incubated with BCG. (XLSX 62 kb) Additional file 4: Table S3. Genes differentially expressed between cluster 1 and cluster 2 infants from the secondary cohort. Gene expression was calculated by subtracting expression in PBMC incubated with medium alone from expression in PBMC incubated with BCG. (XLSX 358 kb) Additional file 5: Table S4. GSEA analysis of differentially expressed genes in cluster 1 and cluster 2 infants from the primary cohort. Gene expression was calculated by subtracting expression in PBMC incubated with medium alone from expression in PBMC incubated with BCG. (XLSX 58 kb) Additional file 6: Table S5. Unique and shared gene expression pathways in each of four groups from the primary cohort: a group of cases and controls within each of the two clusters. Gene expression was from PBMC incubated with medium alone. (XLSX 22 kb) Additional file 7: Table S6. Unique and shared gene expression pathways in each of four groups from the validation cohort: a group of cases and controls within each of the two clusters. Gene expression was from PBMC incubated with medium alone. (XLSX 55 kb)

## Abbreviations

BCG: Bacille Calmette–Guérin
EGF: Epidermal growth factor
ES: Enrichment score
FC: Fold change
FDR: False discovery rate
G-CSF: Granulocyte-colony stimulating factor
GM-CSF: Granulocyte-macrophage colony-stimulating factor
GSEA: Gene Set Enrichment Analysis
IFN-γ: Interferon-gamma
IL: Interleukin
IP-10: 10 kDa interferon gamma-induced protein
IQR: Interquartile range
MAD: Median absolute deviation
MCP-1: Monocyte chemoattractant protein-1
MIP: Macrophage inflammatory protein
MOI: Multiplicity of infection
PBMC: Peripheral blood mononuclear cells
RCT: Randomized controlled trial
SATVI: South African Tuberculosis Vaccine Initiative
sCD40L: Soluble CD40 ligand
SEB: Staphylococcal enterotoxin B
TB: Tuberculosis
TGF-α: Transforming growth factor-alpha
TNF-α: Tumor necrosis factor-alpha
VEGF: Vascular endothelial growth factor
