# Supplementary material for: Human newborn bacille Calmette–Guérin vaccination and risk of tuberculosis disease: a case-control study
Source: BMC Med. 2016 May 16;14:76. doi: 10.1186/s12916-016-0617-3 (PMC4869393; doi:10.1186/s12916-016-0617-3)
Supplement: Additional file 1: Figure S1. — Stability of the two major gene expression clusters. Figure S2. Gene expression in cases and controls. Figure S3. BCG-specific induction of soluble cytokine/chemokine production in cases and controls. Figure S4. Frequencies of proliferating BCG-specific T cells in cases and controls. Figure S5. Frequencies of specific T cells expressing cytotoxic molecules in cases and controls. Figure S6. Frequencies of myeloid and lymphoid cellular subsets in cryopreserved PBMC in cases and controls. Figure S7. Gene expression in the validation cohort of probable TB cases and community controls. Figure S8. Clustering of gene expression in unstimulated samples. Figure S9. In vitro modulation of gene expression by BCG. Figure S10. Frequencies of antigen-specific CD4 and CD8 T cells expressing cytotoxic molecules, and of proliferating BCG-specific T cells, in cases of each cluster, and in controls. (PDF 2443 kb) [file 12916_2016_617_MOESM1_ESM.pdf]

Supplementary Figure S1

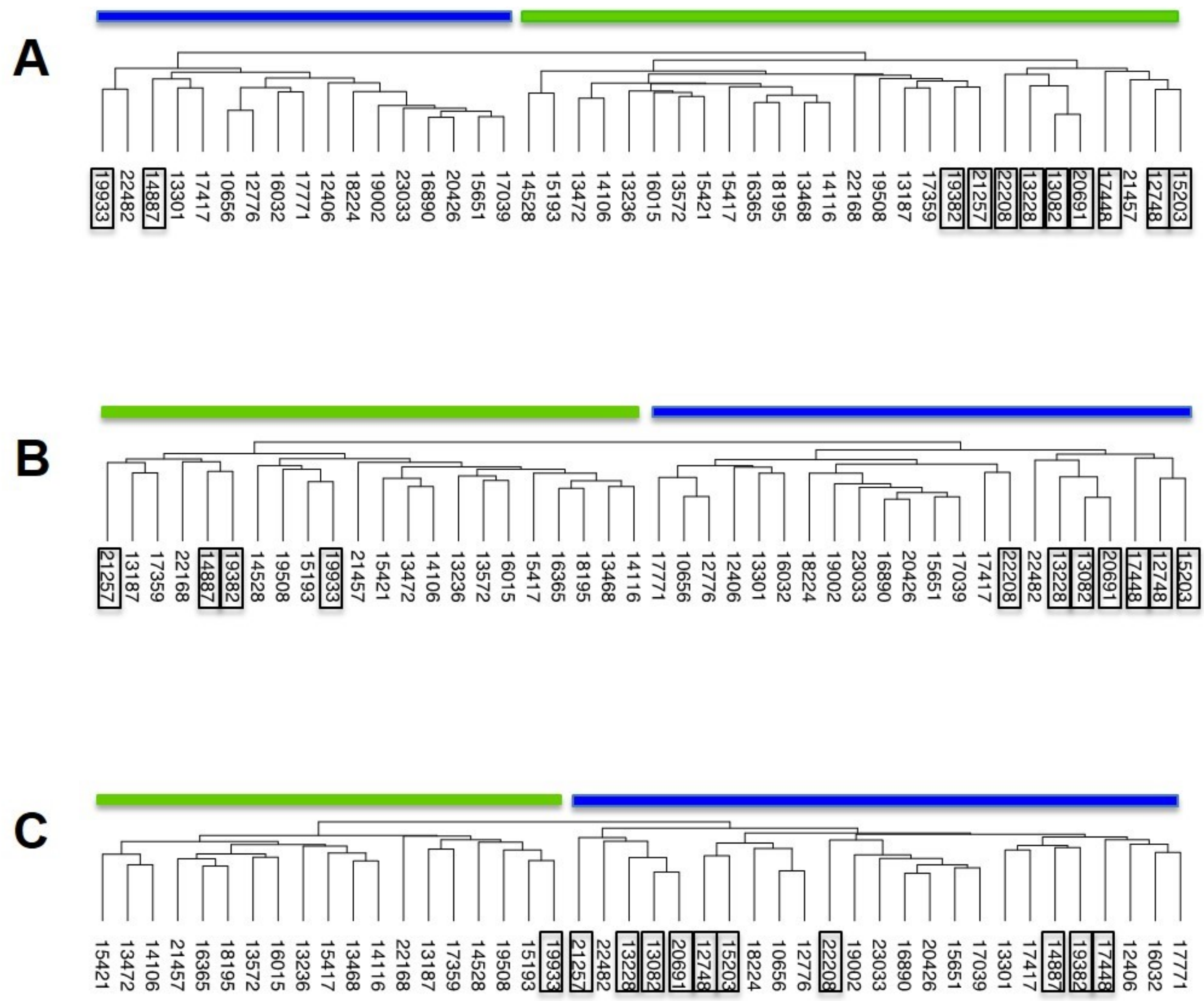

Supplementary figure S1. Stability of the two major gene expression clusters. Clustering of BCG-UNS data was performed using Pearson correlation and stability was tested by changing the number of input genes; 3,077 genes were used in (A), 7902 genes in (B) and 9878 genes in (C). Cluster 1 is shown in blue and cluster 2 in green. There were only 11 infants (black boxes) with an unstable cluster membership, as they switched membership from cluster 1 to cluster 2, dependent upon input gene number. To determine if these infants could be assigned to one of the two major cluster groups, we used pamr and Knn (k=3), a class prediction tool implemented in R, to identify genes able to discriminate between the two major cluster groups of infants and to perform a class prediction for cluster membership of the 11 unstable infants. Pamr and Knn assigned all infants to one of the two major cluster groups (shown in Figure 2).

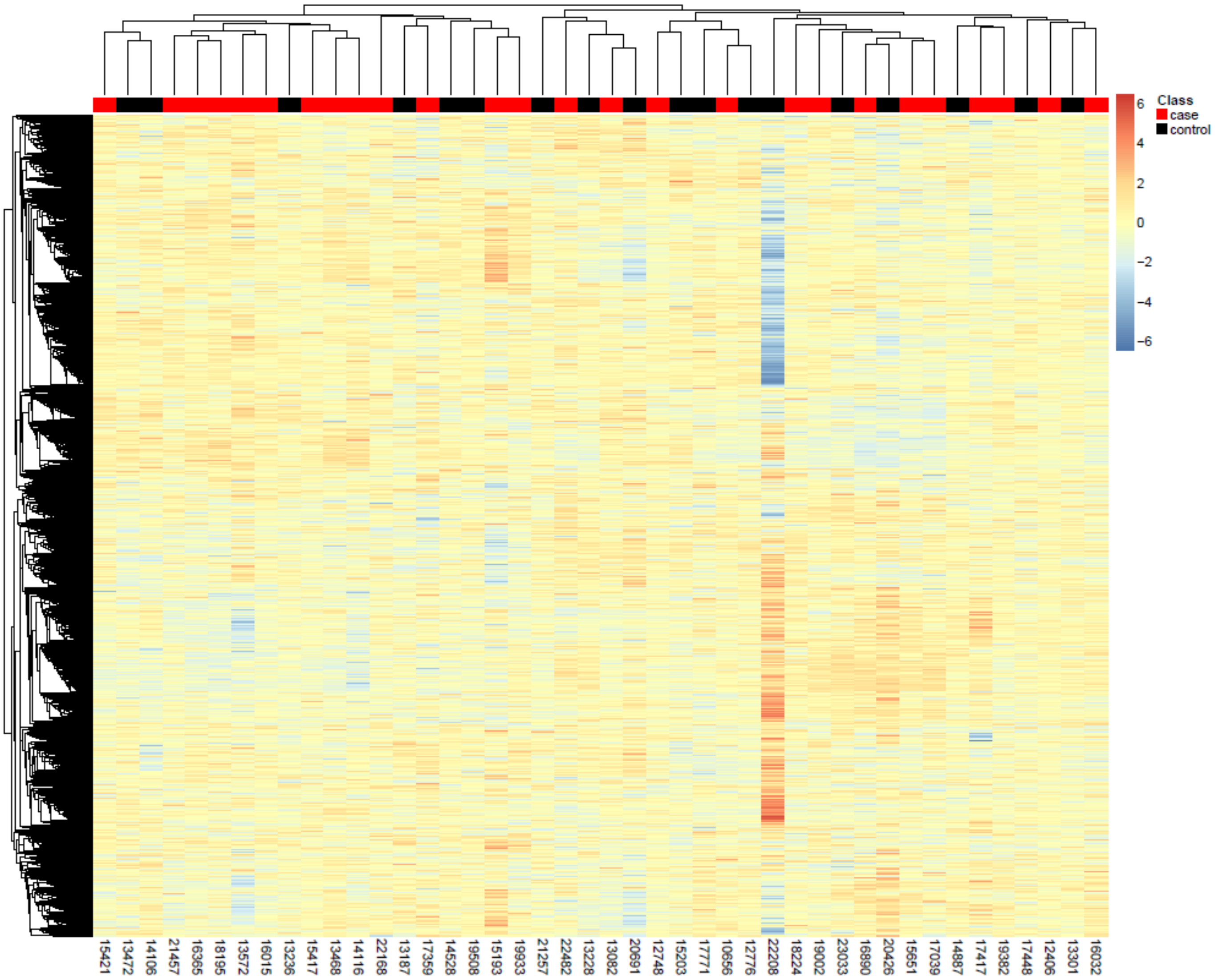

Supplementary figure S2. Gene expression in definite cases (red) and household contact controls (black). The normalized probe intensity obtained from PBMC stimulated with media only was subtracted from the normalized probe intensity obtained from PBMC stimulated with BCG for 12 hours. Using the resulting data set, unsupervised hierarchical clustering was performed (Pearson correlation); no clustering within cases and controls could be shown.

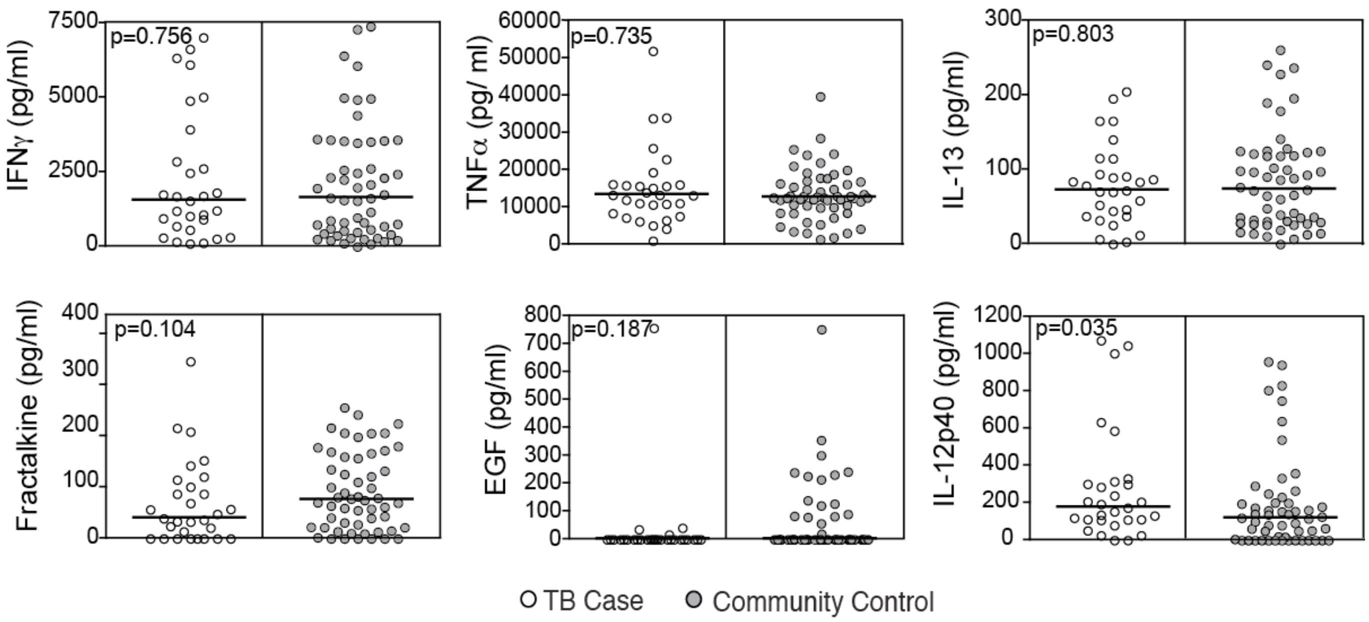

Supplementary figure S3. BCG-specific induction of soluble cytokine/chemokine production in plasma. Whole blood from definite TB cases (n=29) and community controls (n=55) was incubated with BCG and anti-CD28 and anti-CD49d (costimulatory antibodies) for 7 hours, and levels of 29 cytokines/chemokines measured in plasma, by multiplex bead array. BCG-specific levels were calculated by subtracting levels in plasma from whole blood incubated with costimulatory antibodies alone from those in BCG-stimulated blood. Among all 29 cytokines/chemokines, only IL-12p40 levels were different between cases and controls; selected cytokines are shown: IFN-γ, TNFα, IL-13, fractalkine, epidemic growth factor (EGF) and IL-12p40. Horizontal bars represent the median. Groups were compared using the Mann-Whitney U test and unadjusted p-values are shown.

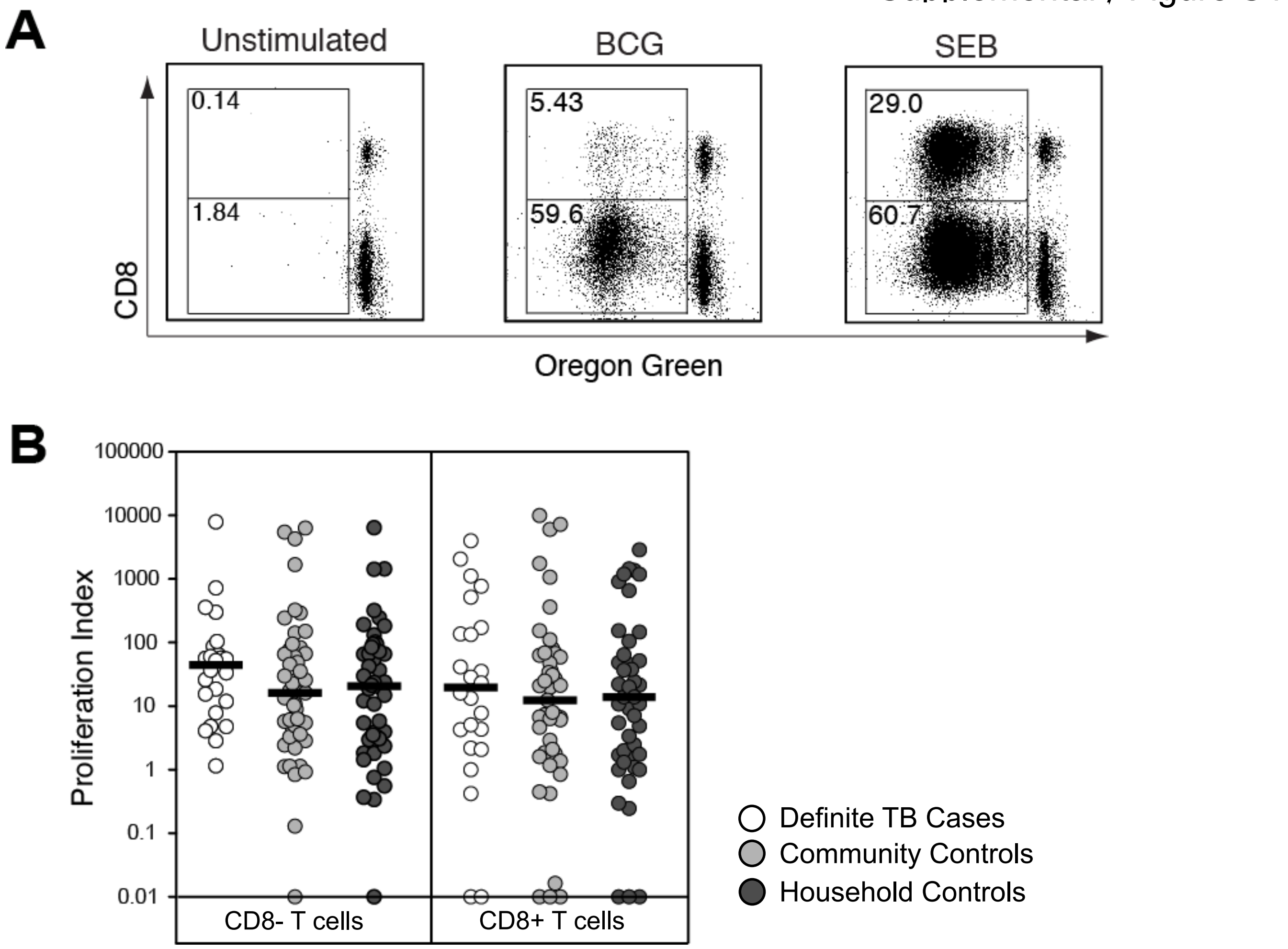

Supplementary figure S4. Frequencies of proliferating BCG-specific T cells. (**A**) Example from a single participant whose PBMC were stained with Oregon Green and incubated for 6 days with either medium alone (Unstimulated), BCG or staphylococcal enterotoxin B (SEB). Proliferating cells were identified by low Oregon Green expression with flow cytometry. Numbers denote the proportion of live CD8+ T cells (top row) or CD8– T cells (bottom row, mostly CD4+ T cells) with low Oregon Green expression. (**B**) BCG-induced proliferating T cells as measured by a proliferation index, i.e., frequency of proliferating cells upon BCG stimulation divided by this frequency in unstimulated cells. Definite TB cases (n=29), household controls (n=55) and community controls (n=55) were included in this analysis. Bars denote medians. The Kruskal-Wallis test was used to compare the three groups, and the Mann-Whitney test was used to compare individual groups. P-values were adjusted for multiple comparisons using the Bonferroni correction (multiplied by 3).

Supplementary Figure S5

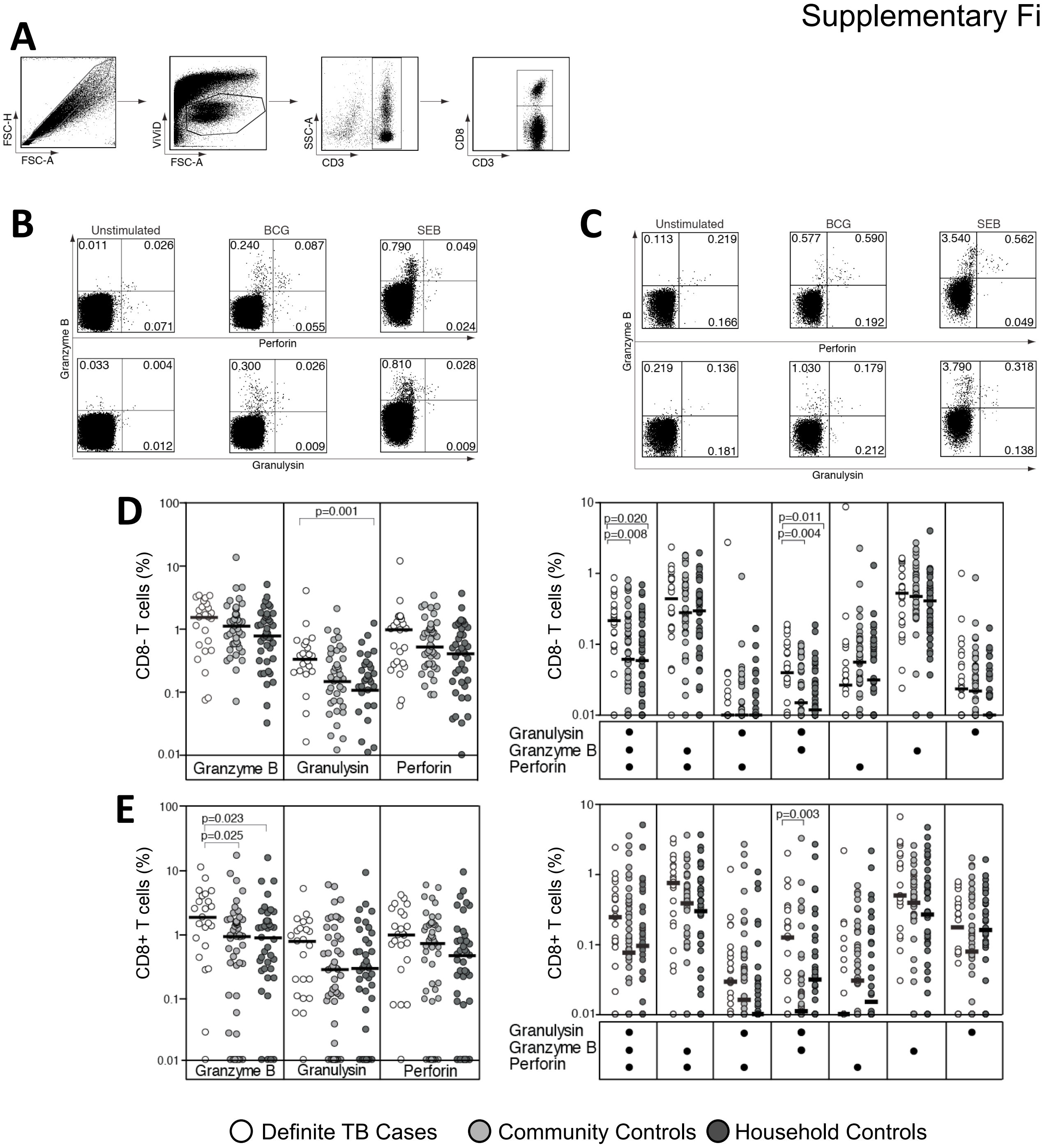

Supplementary figure S5. Frequencies of specific T cells expressing cytotoxic molecules. (**A**) Example from a single participant whose PBMC were incubated for 3 days with either medium alone (Unstimulated), BCG or staphylococcal enterotoxin B (SEB). Intracellular expression of granzyme B, perforin and granulysin by T cells in live cells was detected by flow cytometry. Expression of these markers in CD8- T cells – predominantly CD4+ T cells (**B**) and in CD8+ T cells (**C**) is shown. (**D and E**) Frequencies of total BCG-specific CD8- (**D**) or CD8+ (**E**) T cells expressing granzyme B, granulysin or perforin and their combinations in definite TB cases (n=29), household (n=55) and community controls (n=55). Horizontal lines indicate medians. Groups were compared using the Mann-Whitney test. Only p-values below 0.05 are shown; these were not adjusted for multiple comparisons.

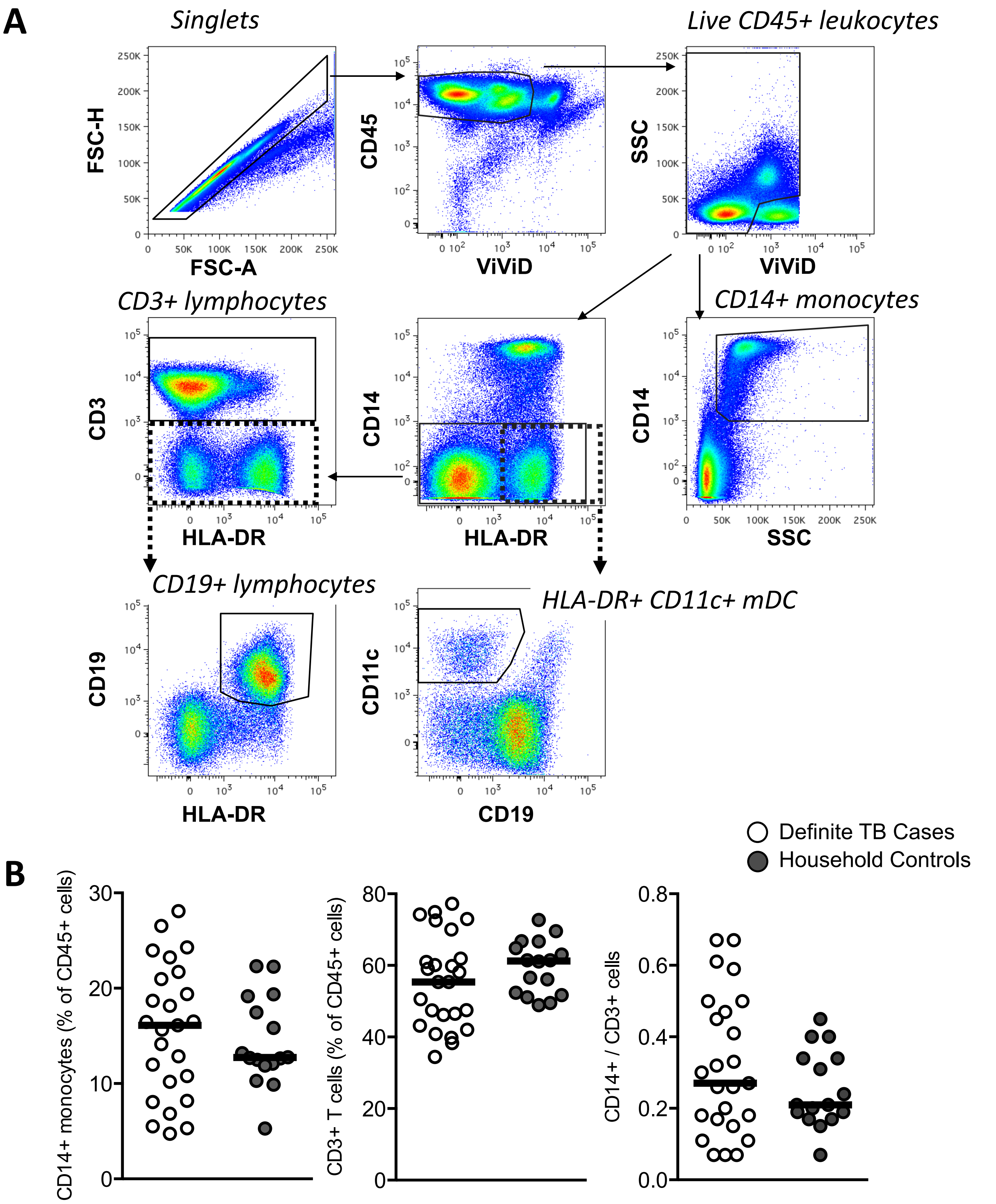

Supplementary figure S6. Frequencies of myeloid and lymphoid cellular subsets in cryopreserved PBMC in definite cases and household controls. (**A**) The gating strategy is shown. Monocytes were defined as CD14+, myeloid dendritic cells were defined as CD14-, CD19-, HLA-DR+ and CD11c+, and lymphocytes were defined as CD3+ or CD19+ cells. Only viable (ViViD^{low}) CD45+ leukocytes were included in analysis. (**B**) No difference in myeloid and lymphoid cell subsets were detected in definite TB cases (n=25) and household controls (n=16): two subset calculations are shown, as well as the ration between monocytes and lymphocytes. Horizontal lines indicate medians. Groups were compared using the Mann-Whitney test and p-values were not adjusted for multiple comparisons.

Supplementary Figure S7

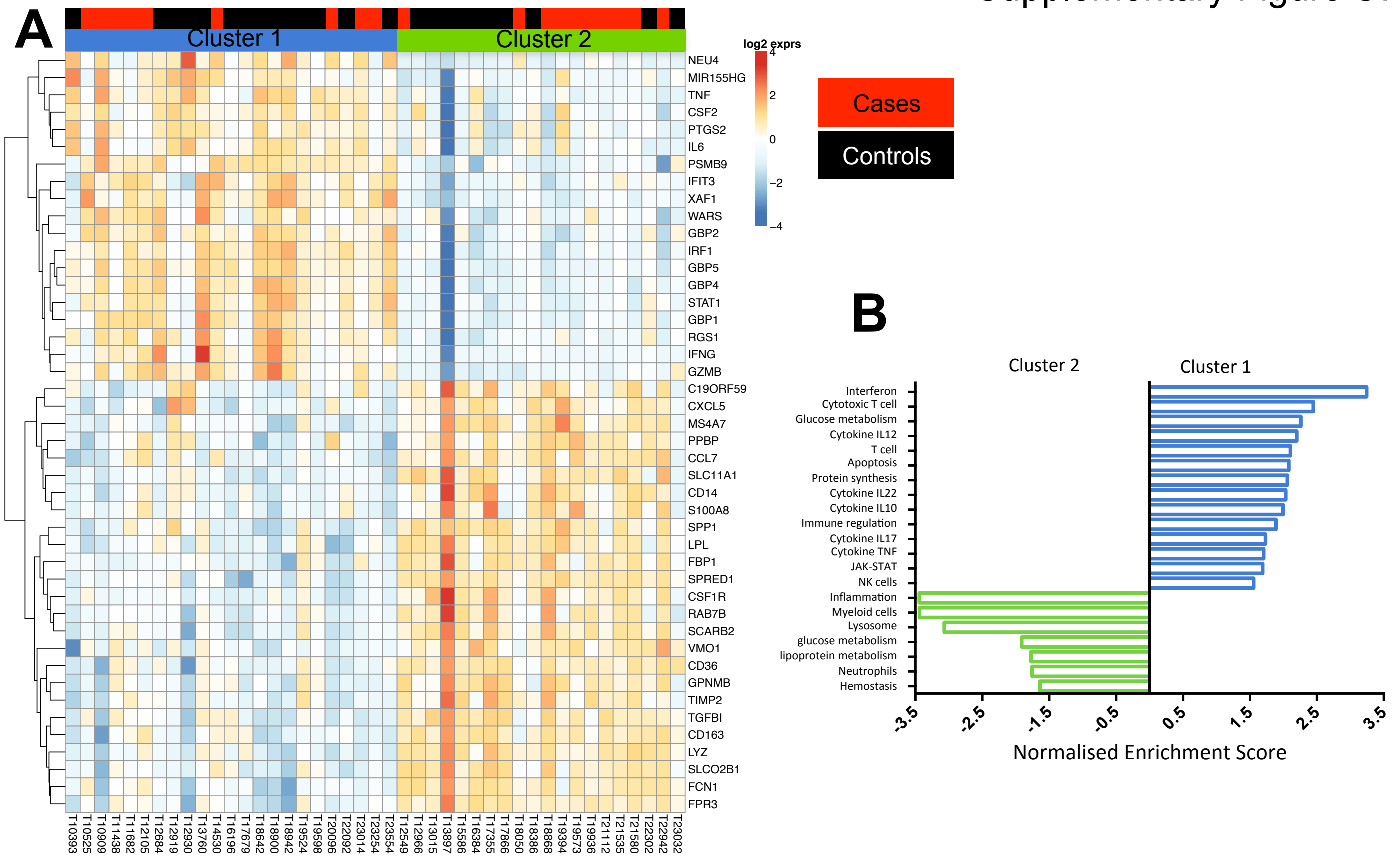

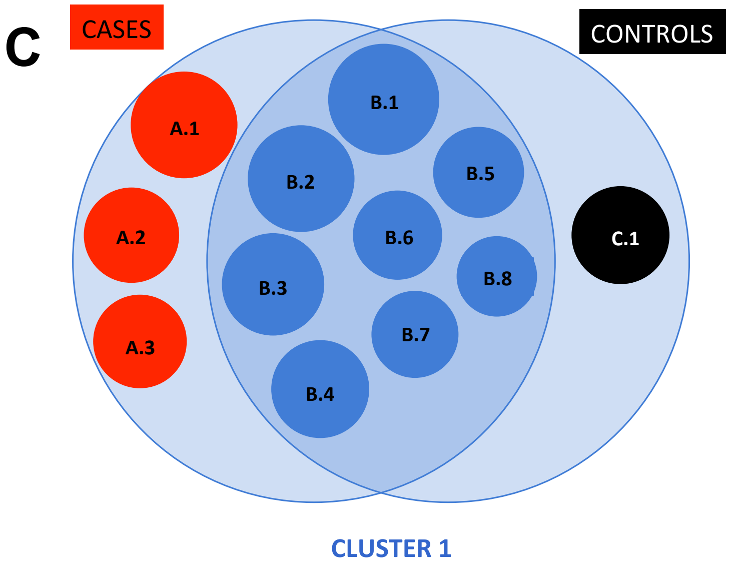

Pathway	Case/control	Description	Gene Set	NES
A.1	case	Platelets	REACTOME_FURTHER_PLATELET_RELEASATE	1.9
A.2	case	AMPK	REACTOME_NUCLEAR_EVENTS_KINASE_AND_TRANSCRIPTION_FACTOR_ACTIVATION	1.8
A.3	case	Fatty acid metabolism	KEGG_TERPENOID_BACKBONE_BIOSYNTHESIS	1.7
B.1	case/control	IFN	M_3.1_INTERFERON_INDUCIBLE	2.5
B.2	case/control	Translation	KEGG_SPLICEOSOME	2.4
B.3	case/control	B cells	M_1.3_B CELLS	2.3
B.4	case/control	Proteasome	KEGG_PROTEASOME	2.2
B.5	case/control	T cells	M_3.8_CDC_TCR_CREB_GLYCOSYLASE	2.04
B.6	case/control	TCA	KEGG_CITRATE_CYCLE_TCA_CYCLE	2
B.7	case/control	Myeloid cells	KEGG_CHRONIC_MYELOID_LEUKEMIA	1.95
B.8	case/control	mTor	BIOCARTA_MTOR_PATHWAY	1.8
C.1	control	Glucose metabolism	KEGG_TYPE_I_DIABETES_MELLITUS	2.2

Supplementary figure S7. Gene expression analysis in the validation cohort of probable TB cases and community controls. (A) Using genes significantly differentially expressed between cluster 1 and cluster 2 infants in our primary cohort of definite TB cases and household contacts, we were able to identify 2 major clusters of infants in this additional cohort. The heatmap shows the top 50 genes differentially expressed between cluster 1 and cluster 2 infants (FDR<5%) upon PBMC stimulation with BCG. **(B)** Biological pathways differentially expressed between cluster 1 and cluster 2 infants from the independent cohort. As observed in the primary cohort, T cell, interferon, cytokine and JAK/STAT signaling were enriched in cluster 1 infants, while myeloid cells and inflammation were enriched in cluster 2 infants. **(C)** In a one-versus-all GSEA analysis of unstimulated PBMC, we identified gene sets uniquely expressed in case (red) and control (black) infants and shared gene sets (Supplementary table 5). The Venn diagram shows unique and shared gene sets sized by NES in cluster 1 infants. As observed in the primary cohort, myeloid and inflammatory pathways were shared among cluster 1 case and control infants. Also in accordance with the primary cohort, glycolysis was enriched in control infants and platelets and AMPK enriched in case infants. There were very few significant gene sets enriched in cluster 2 infants (Supplementary table 5). Unfortunately, only gene expression profiles were assessed in the test cohort, therefore functional and phenotypic outcomes could not be confirmed.

Supplementary Figure S8

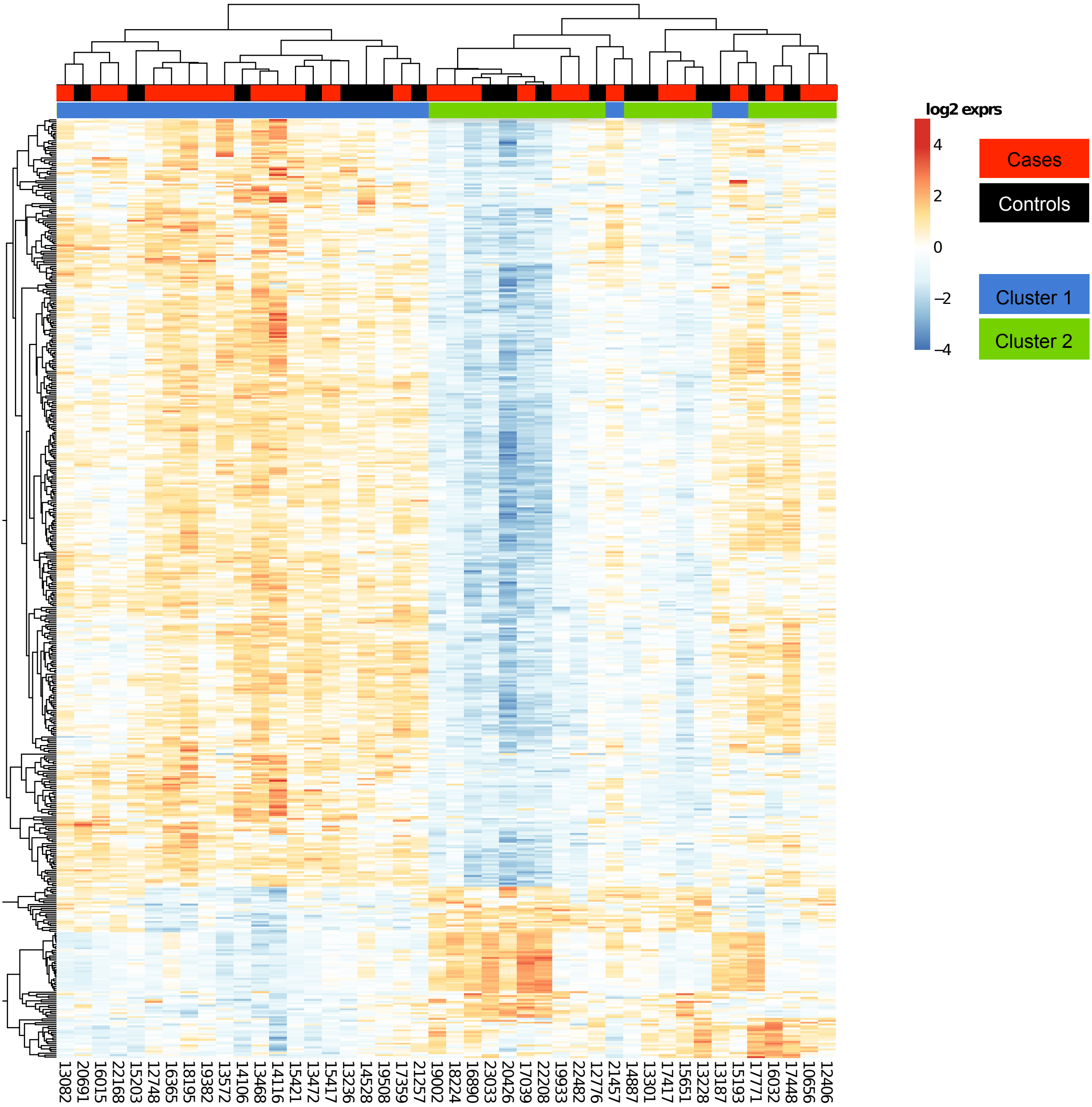

Supplementary figure S8. Clustering of gene expression when unstimulated samples were examined. Samples from the primary cohort of definite cases and household contacts were examined together. The normalized probe intensity obtained from PBMC stimulated with media only was used. Using the resulting data set, unsupervised hierarchical clustering was performed (Pearson correlation), resulting in two major clusters of infants. Overlap with clustering obtained when gene expression of PBMC stimulated with media only was subtracted from that of PBMC stimulated with BCG for 12 hours is shown (Figure 2) – this cluster 1 is marked blue and cluster 2 is marked green. Cases are shown in red, while controls are shown in black.

Supplementary Figure S9

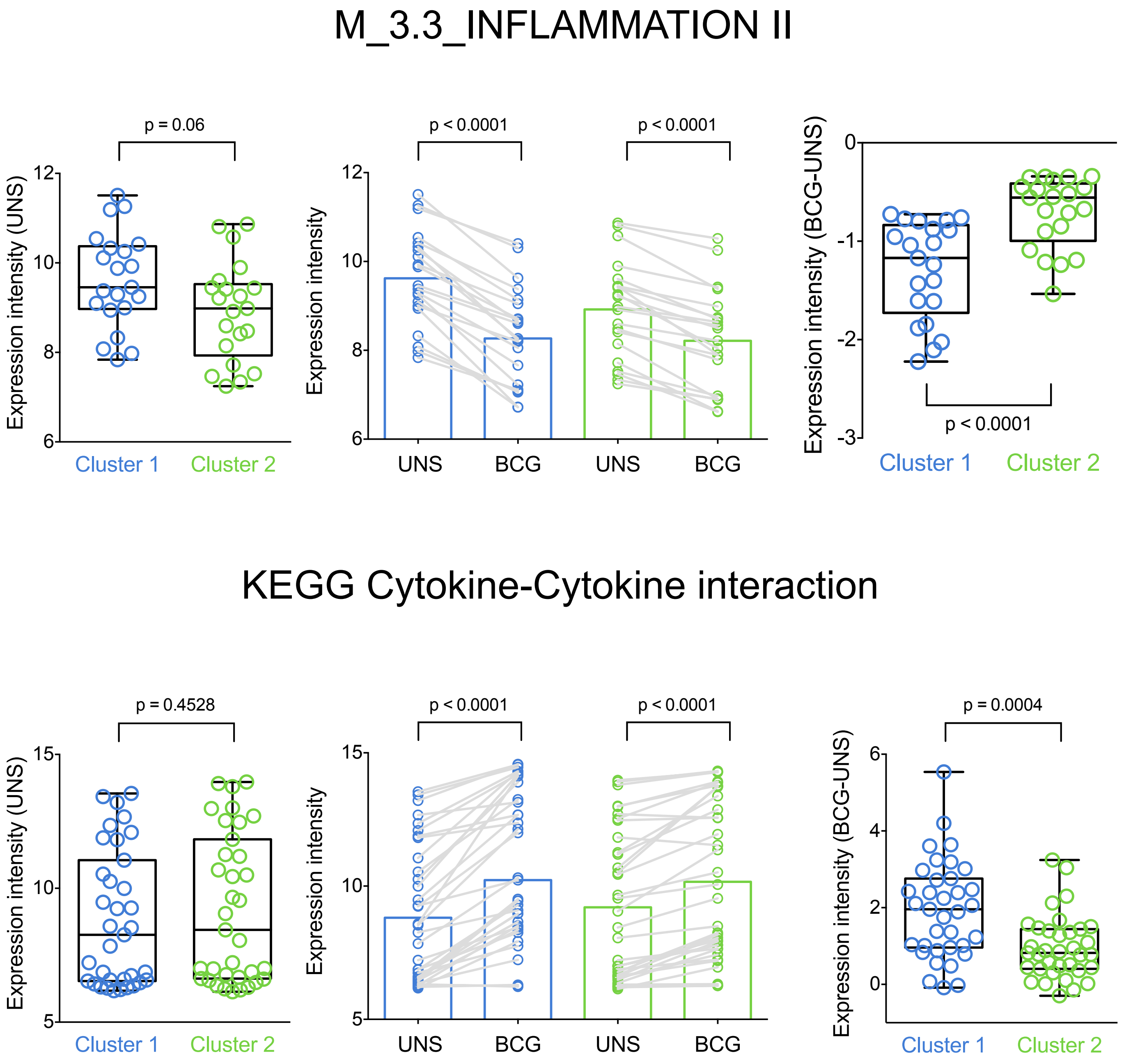

Figure S9. BCG antigen stimulation reduces the expression of inflammatory genes and increases the expression of cytokine genes to a greater extent in cluster 1 infants when compared to cluster 2 infants.
Cluster 1 infants (blue) have higher expression of inflammatory genes in resting (UNS) PBMC compared to cluster 2 infants (green). The expression of these genes is reduced upon BCG—antigen stimulation. As the expression of inflammatory genes is reduced the expression of cytokine specific genes increases, indicating a reciprocal relationship between inflammatory and cytokine pathways in BCG-stimulated infant PBMC. The gene expression intensities of genes differentially expressed between cluster 1 and cluster 2 infants (sup table 1) and present in the GSEA gene sets for M_3.3_INFLAMMATION II and KEGG CYTOKINE-CYTOKINE INTERACTION were plotted for cluster 1 and cluster 2 infants. Box and whiskers plots show medians and interquartile ranges, clusters were compared by Mann-Whitney test. In the middle plots, bars denote median response and P values were determined using a paired T Test.

Supplementary Figure S10

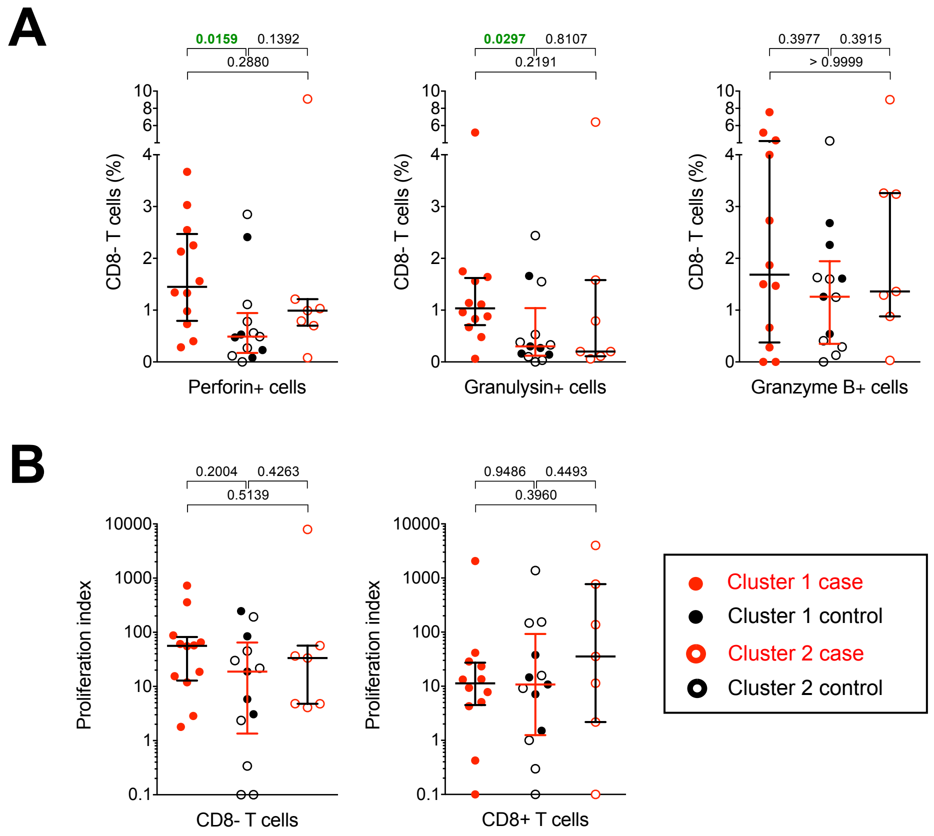

Supplementary figure S10. Frequencies of antigen-specific CD4 and CD8 T cells expressing cytotoxic molecules, and of proliferating BCG-specific T cells, in cases of each cluster, and in controls. (A) To assess expression of cytotoxic molecules, PBMC were processed and analysis completed as described in Supplementary figure S5. Results shown are for CD8- T cells, i.e., predominantly CD4+ T cells. **(B)** To assess proliferation, PBMC were processed and analysis completed as described in Supplementary figure S4. The proliferation index of BCG-specific CD8- and CD8+ T cells was calculated by dividing the frequency of proliferating cells upon BCG stimulation by the frequency in unstimulated cells. For analyses, definite TB case infants from cluster 1 (red closed circles, n=12) and from cluster 2 (red open circles, n=7) were compared to pooled household controls (black closed circles for cluster 1, n=5, and black open circles for cluster 2, n=8). Bars depict medians and interquartile range; the Mann Whitney test was used to assess differences in all analyses. Results from some participants were changed from zero to 0.1 to allow plotting on the logarithmic scale.
